# Preoperative systemic immune-inflammation index as a predictor of contrast-induced acute kidney injury in coronary artery disease: a multicenter cohort study

**DOI:** 10.1080/0886022X.2025.2474204

**Published:** 2025-03-24

**Authors:** Jinlong Zhu, Pei Yu, Xiaoying Zhang, Xiaoming Li, Jiaming Huang, Shumin Zhao, Qingyan Ruan, Yibo He, Yang Zhou, Kunming Bao, Jiaming Xiu, Lin Deng, Yunchen Liu, Yong Liu, Shiqun Chen, Kaihong Chen, Liling Chen

**Affiliations:** a Department of Cardiology, Longyan First Affiliated Hospital of Fujian Medical University, Longyan, China; b The Third Clinical Medical College, Fujian Medical University, Fuzhou, China; c Department of Guangdong Provincial Key Laboratory of Coronary Heart Disease Prevention, Guangdong Cardiovascular Institute, Guangdong Provincial People’s Hospital (Guangdong Academy of Medical Sciences), Southern Medical University, Guangzhou, China; d Global Health Research Center, Guangdong Provincial People’ s Hospital, Guangdong Academy of Medical Science, Southern Medical University, Guangzhou, China

**Keywords:** Acute kidney injury, contrast-induced acute kidney injury, systemic immune-inflammation index, coronary artery disease, nonlinear relationship, biomarker

## Abstract

**Background:**

Inflammation is a key contributor to contrast-induced acute kidney injury (CI-AKI), yet its predictive role remains unclear. The systemic immune-inflammation index (SII) is a novel inflammatory biomarker, but its association with CI-AKI risk in coronary artery disease (CAD) patients undergoing coronary angiography is not well established. This study aimed to evaluate the relationship between preoperative SII and CI-AKI in a large multicenter cohort.

**Methods:**

This retrospective cohort study analyzed CAD patients from five tertiary hospitals in China (2007–2020). Patients were stratified into SII tertiles, and multivariable logistic regression, restricted cubic splines (RCS), and two-piecewise logistic regression models were employed to assess the association between SII and CI-AKI risk.

**Results:**

Among 30,822 patients, 3,246 (10.5%) developed CI-AKI. Higher preoperative SII levels were associated with increased CI-AKI risk ([SII-M vs. SII-L]: OR = 1.22, 95% CI [1.09–1.36], *p* = 0.001; [SII-H vs. SII-L]: OR = 1.70, 95% CI [1.53–1.90], *p* < 0.001). RCS analysis demonstrated a nonlinear relationship (p for nonlinearity = 0.008). The inflection point was at 19.12 × 10^11^/L. Below this inflection point, each 100-unit increase in SII correlated with a 5% higher CI-AKI risk (OR = 1.05, 95% CI [1.04–1.06], *p* < 0.001), while no significant association was observed above this level.

**Conclusion:**

Preoperative SII may be an independent predictor of CI-AKI risk in CAD patients undergoing undergoing coronary angiography, demonstrating a nonlinear dose-response relationship with a significant threshold effect. These findings suggest that SII may serve as a useful biomarker for early CI-AKI risk stratification in clinical practice.

## Introduction

Coronary artery disease (CAD) is one of the leading causes of death globally. In 2016, the global prevalence of CAD was estimated at 154 million, accounting for 32.7% of the worldwide burden of cardiovascular diseases, making it a major cause of mortality[1,2]. Coronary angiography (CAG) is the international gold standard for the diagnosis of coronary heart disease, and percutaneous coronary intervention (PCI) is a recognized effective treatment for coronary heart disease, both of which are commonly employed in clinical practice worldwide. During CAG and PCI procedures, these interventions necessitate the use of contrast agents, which can lead to contrast-induced acute kidney injury (CI-AKI) in the patients undergoing these interventions due to their nephrotoxicity. CI-AKI is the third most common cause of hospital-acquired acute kidney injury[3]. Mehran et al. reported that the prevalence of CI-AKI ranged from 3.3% to 14.5%[4]. Furthermore, CI-AKI is associated not only with adverse clinical outcomes, such as cardiovascular events and chronic kidney disease, but also with progression to chronic renal failure in approximately 25–30% of cases[5]. In addition, CI-AKI significantly prolongs hospital stays and escalates socioeconomic burdens by 5–10 times[6]. CI-AKI severely impacts patients’ health and financial well-being. Despite current preventive measures, such as intravenous hydration, renal replacement therapy (RRT), and pharmacological interventions, their effectiveness remains controversial[4]. Therefore, early identification of high-risk patients and timely interventions are crucial.

Numerous studies have demonstrated that inflammatory markers, including procalcitonin (PCT), C-reactive protein (CRP), high-sensitivity CRP (hs-CRP), neutrophil-to-lymphocyte ratio (NLR), and platelet-to-lymphocyte ratio (PLR), can predict the occurrence of CI-AKI after PCI [7–10]. Although markers like PCT, CRP, and hs-CRP are effective indicators of inflammation, their testing procedures are usually complex and time-consuming, limiting their utility in real-time clinical risk assessment. By contrast, the systemic immune-inflammation index (SII) is more promising due to its easy acquisition and low cost. SII is an inflammatory marker that combines the advantages of NLR and PLR, thereby reflecting both the degree of inflammation and the coagulation pathway [11]. The specific calculation for SII is as follows: SII = neutrophil count × platelet count/lymphocyte count.

However, current studies on the relationship between SII and CI-AKI have primarily focused on patients with acute ST-elevation myocardial infarction (STEMI) [12–14] and the association between SII and the risk of CI-AKI in patients with CAD following CAG remains unclear. Therefore, this study aims to evaluate the relationship between different SII level and the risk of CI-AKI in patients diagnosed with CAD following CAG. This will help improve preoperative risk assessment and reduce the occurrence of CI-AKI.

## Method

### Data sources and patient selection

This multicenter, retrospective study was based on the registry of Cardiorenal ImprovemeNt II (Cardiorenal Improvement II, Clinical-Trials. gov NCT05050877) cohort in five South Chinese regional central tertiary teaching hospitals from 2007 to 2020, which covered 145,267 patients with coronary catheterization.

**Figure 1.**
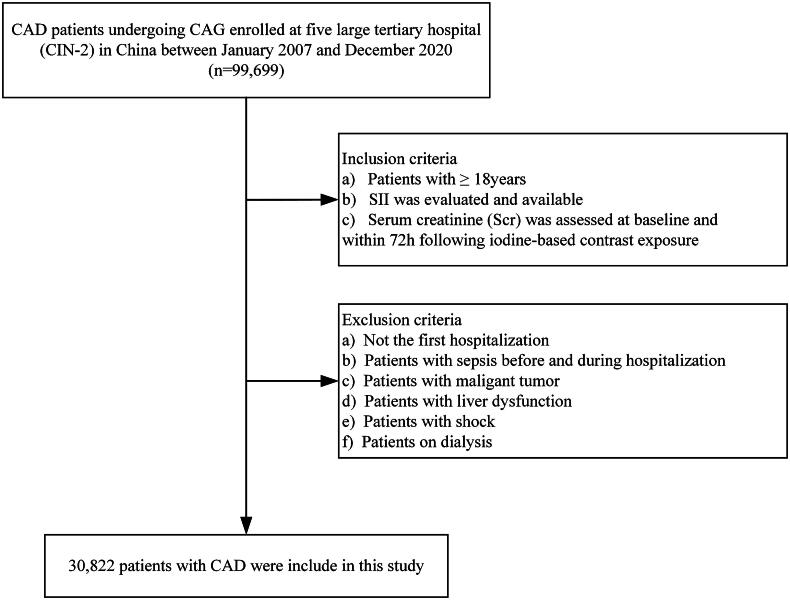
Patient flow diagram.

A group of 99,077 CAD patients diagnosed with CAG during their first hospitalization were included in this analysis. The exclusion criteria were as follows: (1) age < 18 years (*n* = 16); (2) missing neutrophil count, platelet count, or lymphocyte count (*n* = 3,143); (3) missing preoperative serum creatinine (Scr) or postoperative Scr within 72 h (*n* = 62,427); (4) patients with sepsis during admission and hospitalization (*n* = 55); (5) patients with malignant tumors (*n* = 626); (6) patients with abnormal liver function or hepatitis (*n* = 1,071); (7) patients in shock (*n* = 75); (8) patients undergoing dialysis (*n* = 55). Finally, 30,822 participants were included in the study (Figure 1). Personally identifiable information has been removed from the analytical dataset to protect patient privacy. This clinical study was conducted in accordance with the Declaration of Helsinki and approved by the Ethics Committee of Guangdong Provincial People’s Hospital (approval number GDREC2019-555H-2). Ethical approval was also obtained from the institutional review boards and ethics committees of all participating centers. As this study is a retrospective cohort study, informed consent was waived.

### Data collection and measurements

The baseline data was collected from the Electronic Clinical Management System (ECMS) for all participant hospitals, including demographic information, coexisting conditions, laboratory test results, medication lists, and procedural details. Two senior cardiologists were responsible for data quality control and periodic database validation. Biochemistry data, including platelets, neutrophils, and lymphocytes, were measured on admission by an automatic biochemical analyzer before the procedure. The serum creatinine (Scr) concentration was measured by a kinetic colorimetric assay (Jaffe) using the machine (Beckman, AU5800, the United States) at admission and within 72h after administration of contrast. The rest of the biochemical indicator measurement was performed on admission. Uniform methods of measurement were used in all centers.

### Exposure and outcomes

The primary exposure was baseline preoperative SII in all participants, which was calculated as follows: SII = (neutrophil count × platelet count)/lymphocyte count. The primary endpoint was CI-AKI, defined according to the European Society of Urogenital Radiology (ESUR) as an increase in Scr level of ≥ 0.5 mg/dL or 25% from baseline within 72 h after the administration of contrast agents [15]. The estimated glomerular filtration rate (eGFR) was calculated using the Chronic Kidney Disease Epidemiology Collaboration (CKD-EPI) equation [16]. eGFR < 60 mL/min/1.73 m^2^ is defined as CKD. Congestive heart failure (CHF) is defined as New York Heart Association Grade > 2 or Killip grade > 1 [17]. According to the World Health Organization (WHO) criteria, anemia was defined as a hematocrit level ≤ 39% in men or ≤ 36% in women (the definition of anemia in the CI-AKI risk score developed by Mehran et al. [18] was used in this study). Diabetes mellitus (DM), STEMI, hypertension, hypotension, hyperuricemia, and stroke were defined using ICD-10 codes.

### Statistical analysis

The study population was divided into three groups based on the tertiles of the SII: low SII group (SII-L), medium SII group (SII-M), and high SII group (SII-H). The distribution of each variable was evaluated by the Kolmogorov–Smirnov test. Normally distributed variables are reported as mean ± SDs, while variables with skewed distribution are reported as median (interquartile interval). Categorical variables were reported as frequencies and percentages. Differences of groups in baseline characteristics were compared through the use of Student’s t-test and ANOVA for continuous variables and chi-square tests for categorical variables. Multivariate logistic regression analysis was performed to examine the relationship between the SII and CI-AKI. Odds ratios (OR) and 95% confidence intervals (CI) were used to evaluate the association between SII and CI-AKI in each model.

Furthermore, restricted cubic spline (RCS) analysis was employed to assess potential nonlinear relationships between SII and CI-AKI. Subsequently, a smooth fitting curve was generated using the generalized additive model (GAM) to visually present the relationship between SII level and CI-AKI. Following previous studies [19,20], we determined the inflection point by using the R package ‘segmented’. The standard linear model and two-piecewise linear model were applied to examine which model was the better one for fitting the relationship between SII level and CI-AKI. The best-fitting model was confirmed based on the P-value of the log-likelihood ratio test. If the P-value < 0.05, the two-piecewise linear model was better. If the P-value ≥ 0.05, the standard linear model was better. We also used the threshold effect analysis model by building a two-piecewise logistic regression model to evaluate the effect sizes and confidence intervals on both sides of the inflection point.

Subgroup analyses were performed to explore potential effect modifiers, stratifying by age, gender, eGFR, CHF, AMI, DM, PCI, and hypertension. Statistical significance was set at a two-tailed P value < 0.05. All analyses were conducted using R version 4.3.1 (R Foundation for Statistical Computing, Vienna, Austria).

## Result

### Baseline characteristics

A total of 30,822 patients were included in the study and divided into three groups according to SII tertiles: the SII-L group (SII < 3.74 × 10^11^/L, *n* = 10,274), SII-M group (3.74 × 10^11^/L ≤ SII < 6.27 × 10^11^/L, *n* = 10,274), and SII-H group (SII ≥ 6.27 × 10^11^/L, *n* = 10,274). The average age of the study population was 63.7 ± 11.3 years, with 23,513 (76.3%) male patients (Table 1). Patients with elevated SII exhibited significantly elevated levels of hypersensitive C-reactive protein (hs-CRP), cholesterol (CHOL), low-density lipoprotein cholesterol (LDL-C), neutrophil count, platelet count, monocyte count, and lymphocyte count (all *p* < 0.05). In contrast, they had lower values for estimated glomerular filtration rate (eGFR), triglycerides (TRIG), hemoglobin (HGB), albumin (ALB), and left ventricular ejection fraction (LVEF) (all *p* < 0.05). Additionally, CAD patients with higher SII level were more likely to have STEMI (9.0% vs. 20.8% vs. 44.5%, *p* < 0.001), hypertension (55.9% vs. 59.9% vs. 56.3%, *p* < 0.001), congestive heart failure (13.3% vs. 18.3% vs. 29.0%, *p* < 0.001), chronic kidney disease (20.3% vs. 25.5% vs. 29.6%, *p* < 0.001), anemia (30.1% vs. 34.1% vs. 38.3%, *p* < 0.001), undergoing PCI (73.1% vs. 77.5% vs. 83.2%, *p* < 0.001), and receiving intra-aortic balloon pump (1.7% vs. 2.4% vs. 5.6%, *p* < 0.001). Patients in the SII-M group had a significantly higher proportion of DM than the other two groups (*p* < 0.05). There were no statistically significant differences among the three groups regarding hyperuricemia (36.1% vs. 35.5% vs. 35.9%, *p* = 0.706).

**Table 1.** Baseline characteristics of the patients in different SII groups.

Characteristics	Overall	Tertile of systemic immune inflammation index			*P*-value
		SII-L	SII-M	SII-H	
	(*n* = 30,822)	(*n* = 10,274)	(*n* = 10,274)	(*n* = 10,274)	
Demographic characteristics					
Age, years, mean (SD)	63.7 (11.3)	63.6 (10.7)	63.8 (11.1)	63.7 (12.2)	0.487
Female, *n* (%)	7,309 (23.7)	2,601 (25.3)	2,431 (23.7)	2,277 (22.2)	<0.001
Age >75, *n* (%)	9,196 (29.8)	2,857 (27.8)	3,060 (29.8)	3,279 (31.9)	<0.001
SBP, mmHg, mean (SD)	131.7 (21.3)	132.0 (19.9)	133.1 (21.3)	129.4 (22.8)	<0.001
DBP, mmHg, mean (SD)	75.9 (11.5)	75.9 (11.0)	76.3 (11.4)	75.2 (12.2)	<0.001
Smoke, *n* (%)	6,477 (29.1)	2,097 (27.2)	2,152 (28.9)	2,228 (31.6)	<0.001
Drink, *n* (%)	1,904 (8.6)	649 (8.4)	634 (8.5)	621 (8.8)	0.695
Comorbidities					
STEMI, *n* (%)	7,645 (24.8)	928 (9.0)	2,142 (20.8)	4,575 (44.5)	<0.001
Hypertension, *n* (%)	17,684 (57.4)	5,748 (55.9)	6,152 (59.9)	5,784 (56.3)	<0.001
Hypotension, *n* (%)	1,110 (6.4)	364 (5.5)	354 (5.9)	392 (8.4)	<0.001
DM, *n* (%)	11,095 (36.0)	3,691 (35.9)	3,838 (37.4)	3,566 (34.7)	<0.001
CHF, *n* (%)	6,228 (20.2)	1,369 (13.3)	1,882 (18.3)	2,977 (29.0)	<0.001
CKD, *n* (%)	7,754 (25.2)	2,088 (20.3)	2,620 (25.5)	3,046 (29.6)	<0.001
Anemia, *n* (%)	10,530 (34.2)	3,093 (30.1)	3,507 (34.1)	3,930 (38.3)	<0.001
Hyperuricemia, *n* (%)	8,803 (35.8)	3,010 (36.1)	2,979 (35.5)	2,814 (35.9)	0.706
PCI, *n* (%)	24,022 (77.9)	7,515 (73.1)	7,963 (77.5)	8,544 (83.2)	<0.001
IABP, *n* (%)	994 (3.2)	177 (1.7)	243 (2.4)	574 (5.6)	<0.001
Laboratory tests					
eGFR, mL/min/1.73m^2^, mean (SD)	76.6 (26.4)	78.9 (24.6)	76.3 (26.4)	74.5 (28.0)	<0.001
HGB, mg/dL, mean (SD)	133.1 (18.0)	134.5 (16.4)	133.1 (17.5)	131.6 (19.8)	<0.001
Scr, mg/dL, mean (SD)	1.0 [0.8, 1.2]	0.9 [0.8, 1.1]	1.0 [0.8, 1.2]	1.0 [0.8, 1.2]	<0.001
HbA1c, %, mean (SD)	6.6 (1.5)	6.6 (1.4)	6.7 (1.5)	6.7 (1.6)	0.006
UA, umol/L, mean (SD)	395.0 (118.6)	395.1 (109.0)	394.5 (117.4)	395.3 (129.0)	0.901
CHOL, umol/L, mean (SD)	4.6 (1.3)	4.5 (1.2)	4.6 (1.2)	4.7 (1.3)	<0.001
TRIG, mmol/L, mean (SD)	1.7 (1.3)	1.8 (1.5)	1.7 (1.3)	1.6 (1.2)	<0.001
LDL-C, mg/dL, mean (SD)	2.9 (1.1)	2.8 (1.0)	2.9 (1.0)	3.1 (1.1)	<0.001
HDL-C, mg/dL, mean (SD)	1.0 (0.3)	1.0 (0.3)	1.0 (0.3)	1.1 (0.3)	<0.001
ALB, g/L, mean (SD)	37.4 (4.8)	38.0 (4.3)	37.4 (4.7)	36.8 (5.3)	<0.001
LVEF, %, mean (SD)	57.3 (12.5)	59.3 (12.3)	57.8 (12.3)	54.7 (12.3)	<0.001
Hs-CRP, mg/L, mean (SD)	13.6 (28.3)	6.0 (13.7)	11.7 (22.0)	23.3 (39.9)	<0.001
Neutrophil,10^9^/L (median [IQR])	5.2 [4.0, 7.4]	3.8 [3.1, 4.5]	5.2 [4.3, 6.3]	8.3 [6.4, 10.6]	<0.001
Lymphocyte,10^9^/L (median [IQR])	1.7 [1.3, 2.2]	2.1 [1.7, 2.5]	1.8 [1.4, 2.2]	1.3 [1.0, 1.7]	<0.001
Monocyte, 10^9^/L (median [IQR])	0.6 [0.4, 0.8]	0.5 [0.4, 0.7]	0.6 [0.5, 0.8]	0.7 [0.5, 0.9]	<0.001
Platelet, 10^9^/L (median [IQR])	221.7 [186.0, 263.0]	197.0 [167.7, 229.0]	226.3 [193.3, 265.3]	246.0 [206.6, 292.0]	<0.001
Discharge medication					
ACEI/ARB, *n* (%)	20,822 (71.4)	7,053 (71.8)	7,116 (73.0)	6,653 (69.4)	<0.001
β-blocker, *n* (%)	23,683 (81.2)	7,822 (79.7)	7,988 (82.0)	7,873 (82.1)	<0.001
Statins, *n* (%)	27,884 (95.7)	9,399 (95.7)	9,314 (95.6)	9,171 (95.6)	0.884
Aspirin, *n* (%)	27,075 (92.9)	9,072 (92.4)	9,036 (92.8)	8,967 (93.5)	0.010
Spironolactone, *n* (%)	5,405 (18.5)	1,280 (13.0)	1,639 (16.8)	2,486 (25.9)	<0.001

Abbreviations: STEMI, ST-elevation myocardial infarction; DM, diabetes mellitus; CHF, congestive heart failure; CKD, chronic kidney disease; PCI, percutaneous coronary intervention; IABP, intra-aortic balloon pump; eGFR, estimated glomerular filtration rate; HGB, hemoglobin; Scr, serum creatinine; HbA1c, glycosylated hemoglobin type A1C; UA, uric acid; CHOL, cholesterol; TRIG, triglycerides; LDL-C, low-density lipoprotein cholesterol; HDL-C, high-density lipoprotein cholesterol; ALB, albumin; LVEF, left ventricular ejection fractions; Hs-CRP, hypersensitive C-reactive protein; ACEI/ARB, angiotensin converting enzyme inhibitors/angiotensin Receptor Blockers.

In terms of medication usage, patients with higher SII level were more likely to use beta-blockers (79.7% vs. 82.0% vs. 82.1%, *p* < 0.001), spironolactone (13.0% vs. 16.8% vs. 25.9%, *p* < 0.001), ACEI/ARB (71.8% vs. 73.0% vs. 69.4%, *p* < 0.001), and aspirin (92.4% vs. 92.8% vs. 93.5%, *p* = 0.010). However, there was no significant difference in statin use among the three groups (95.7% vs. 95.6% vs. 95.6%, *p* = 0.884).

### Association between preoperative SII level and CI-AKI risk

**Figure 2.**
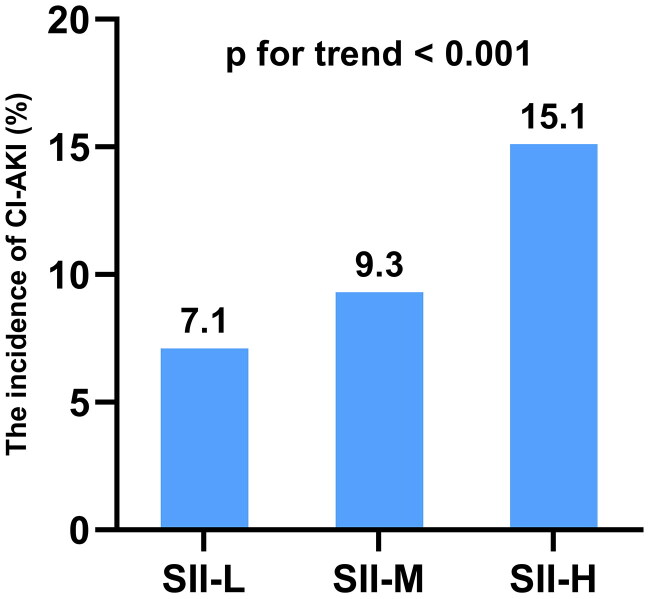
The incidence of CI-AKI in the three groups.

**Figure 3.**
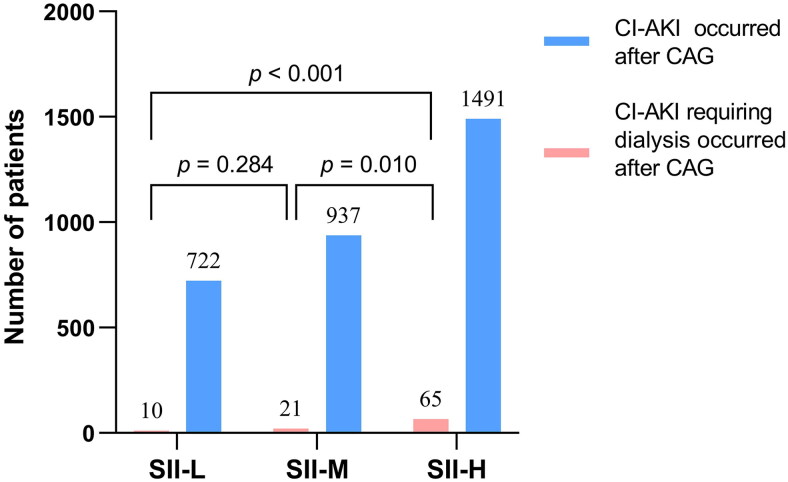
Comparison of the number of patients with CI-AKI and CI-AKI requiring dialysis after CAG according to SII tertiles.

In this study, 3,246 patients (10.5%) developed CI-AKI. The incidence of CI-AKI was 7.1%, 9.3%, and 15.1% in the SII-L, SII-M, and SII-H groups (P for trend < 0.001). The relationship between the three groups and the incidence of CI-AKI is presented in Figure 2. Furthermore, we compared the number of patients with CI-AKI and CI-AKI requiring dialysis after CAG based on the tertiles of SII and showed this in Figure 3. The results showed that there was no statistically significant difference in the number of CI-AKI with dialysis occurrence between the SII-L and SII-M groups (*p* = 0.284). However, there were statistically significant differences between the SII-H group and the SII-L group (*p* < 0.001), and between the SII-H group and the SII-M group (*p* = 0.010), indicating that the risk of occurrence of CI-AKI with dialysis increased significantly with increasing levels of SII.

Logistic regression analysis was conducted to determine the relationship of SII with CI-AKI risk (Table 2). The univariate analysis (Model 1) indicated that patients with elevated SII level were associated with a markedly increased risk of developing CI-AKI in comparison to those categorized in the SII-L group ([SII-M vs. SII-L]: OR = 1.34, 95% CI [1.21–1.48], *p* < 0.001; [SII-H vs. SII-L]: OR = 2.37, 95% CI [2.12–2.55], *p* < 0.001). Model 2 and Model 3 were the multivariable analysis. Model 2 was adjusted for age and gender, and Model 3 was adjusted for age, gender, DM, hypertension, congestive heart failure, hyperuricemia, anemia, low-density lipoprotein cholesterol, IABP, ACEI/ARB, statins, and spironolactone. Following the adjustment for confounding variables, the multivariate analysis (Model 3) continued to demonstrate that the higher SII level was correlated with an increased risk of CI-AKI ([SII-M vs. SII-L]: OR = 1.22, 95% CI [1.09–1.36], *p* = 0.001; [SII-H vs. SII-L]: OR = 1.70, 95% CI [1.53–1.90], *p* < 0.001).

**Table 2.** Univariable and multivariable logistic regression analysis of the association between preprocedural SII level and CI-AKI.

Groups	Event, n(%)	Model 1		Model 2		Model 3	
		OR (95% CI)	*P* value	OR (95% CI)	*P* value	OR (95% CI)	*P* value
SII-L	732 (7.1)	Ref.		Ref.		Ref.	
SII-M	958 (9.3)	1.34 (1.21-1.48)	<0.001	1.35 (1.22-1.49)	<0.001	1.22 (1.09-1.36)	0.001
SII-H	1,556 (15.1)	2.37 (2.12-2.55)	<0.001	2.35 (2.15-2.58)	<0.001	1.70 (1.53-1.90)	<0.001

Model 1: unadjusted.

Model 2: adjusted for age and gender.

Model 3: adjusted for age, gender, DM, hypertension, congestive heart failure, hyperuricemia, anemia, low-density lipoprotein cholesterol, IABP, ACEI/ARB, statins, and spironolactone.

### Nonlinear relationship between SII and CI-AKI

**Figure 4.**
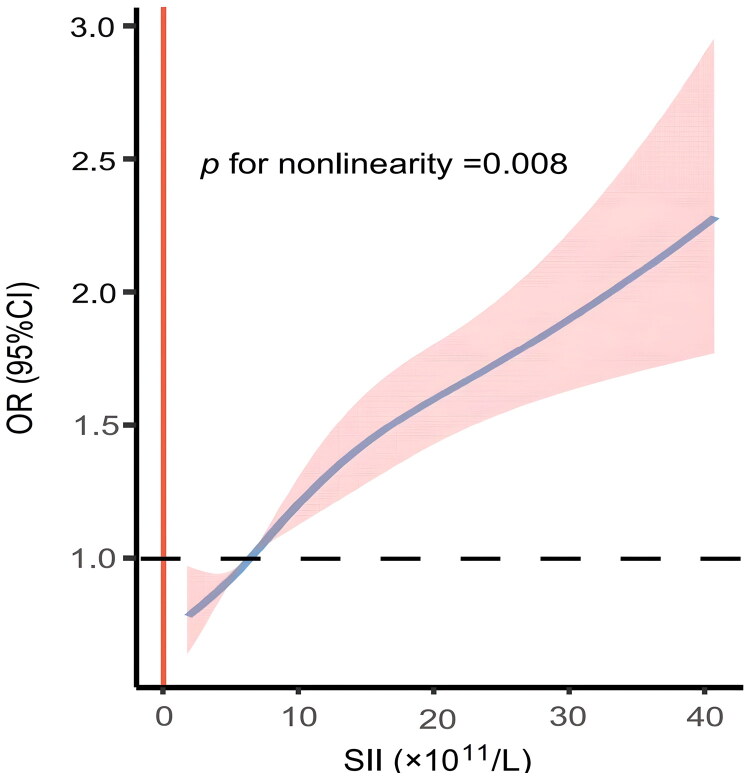
Restricted cubic spline illustrates the relationship between SII and the risk of CI-AKI. Adjusted for age, gender, DM, hypertension, congestive heart failure, hyperuricemia, anemia, low-density lipoprotein cholesterol, IABP, ACEI/ARB, statins, and spironolactone.

A standard logistic regression model revealed that for every 100-unit increase in SII, the adjusted odds ratios for the risk of CI-AKI increased by 3% (OR = 1.03, 95% CI [1.03–1.04], *p* = 0.001). RCS indicated a nonlinear association between SII and CI-AKI risk (P for nonlinearity = 0.008) (Figure 4). The P value for the log-likelihood ratio test was less than 0.001 (Table 3), which indicated that the two-piecewise linear model was the best. We further calculated the inflection point at 19.12 × 10^11^/L. Threshold effects analysis further quantified this nonlinear relationship. Specifically, our analysis showed that for each 100-unit increase in SII below the inflection point (SII ≤ 19.12 × 10^11^/L), the adjusted odds ratios (OR) for the risk of CI-AKI increased by 5% (OR = 1.05, 95% CI [1.04–1.06], *p* < 0.001). Conversely, above the inflection point (SII > 19.12 × 10^11^/L), the increase in SII no longer had a significant effect on the risk of CI-AKI (OR = 1.01, 95% CI [0.99–1.03], *p* = 0.24).

**Table 3.** Threshold effect analysis of SII (per 100-unit increase) on CI-AKI in patients with coronary artery disease.

	Adjusted OR (95% CI), *P* value
Fitting by standard Logistic regression model	1.03 (1.03–1.04) 0.001
Fitting by two-piecewise Logistic regression model	
The inflection point of SII	1,912
SII < 19.12 × 10^11^/L	1.05 (1.04–1.06) 0.001
SII ≥ 19.12 × 10^11^/L	1.01 (0.99–1.03) 0.240
P for Log-likelihood ratio	<0.001

Adjusted for age, gender, DM, hypertension, congestive heart failure, hyperuricemia, anemia, low-density lipoprotein cholesterol, IABP, ACEI/ARB, statins, and spironolactone.

**Figure 5.**
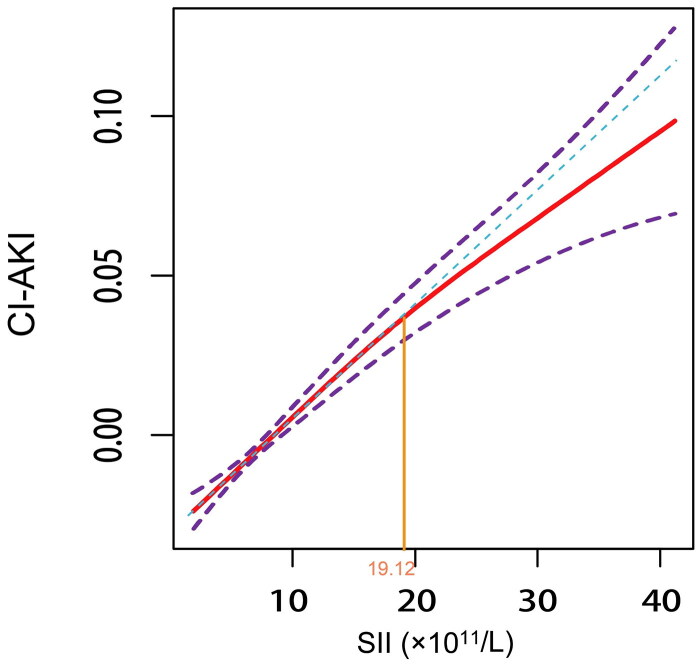
The smooth fitting curve indicated a nonlinear relationship between SII level and CI- AKI. The red solid line represented the smooth fitting curve between SII level and CI-AKI risk, the purple dotted line represented the 95% confidence interval of the fitting curve, and the blue dotted line was a straight reference line, which was intended to provide a visual comparison of the fitting curve and highlighted the location of the inflection point. Adjusted for age, gender, DM, hypertension, congestive heart failure, hyperuricemia, anemia, low-density lipoprotein cholesterol, IABP, ACEI/ARB, statins, and spironolactone. Abbreviations: SII, indicates systemic immune-inflammation index; CI-AKI, contrast-induced acute kidney injury.

The smooth fitting curve for the relationship between SII level and CI-AKI is shown in Figure 5. Although it was close to a linear relationship, we still found that the relationship between the two is nonlinear with the help of the blue reference line. It showed that the risk of CI-AKI increased steadily with increasing SII below the inflection point. Conversely, when SII exceeded the inflection point, the rising trend of CI-AKI risk slowed down, which was consistent with the result of the threshold effect analysis. This finding indicated that when SII was below the inflection point, its ability to assess the early risk of CI-AKI was quite effective.

### Subgroup analysis

**Figure 6.**
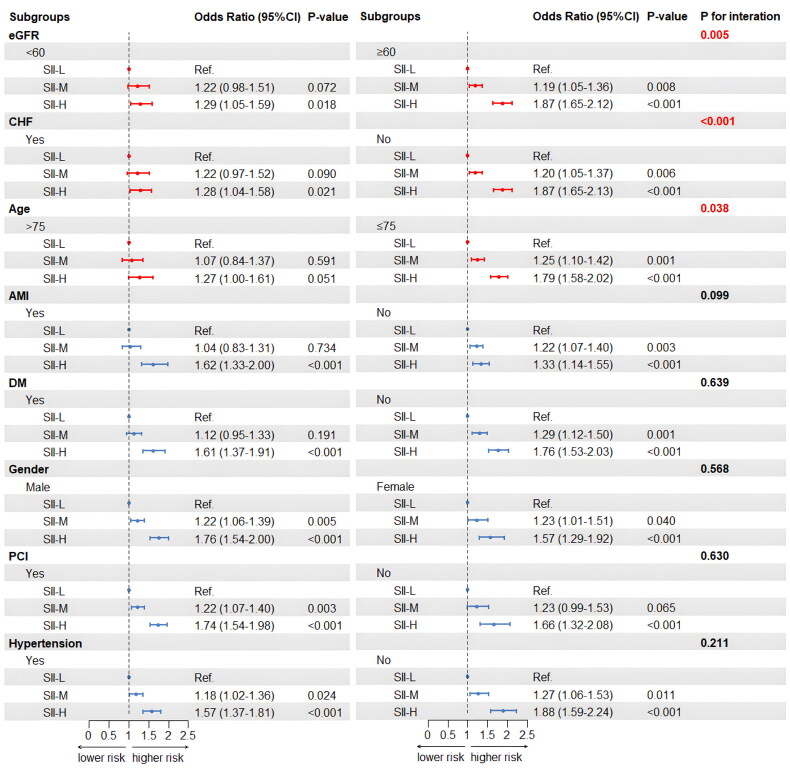
Forest plots of SII for CI-AKI in prespecified subgroups. Patients are dichotomized according to eGFR (< 60 or ≥ 60 mL/min/1.73 m^2^), age (< 75 or ≥ 75 years.), gender (male or female), history of CHF, AMI, DM, PCI, and hypertension (yes or no). Multivariable logistic regression in subgroups adjusted the same covariates of model 3 in Table 2. Abbreviations: SII, systemic immune-inflammation index; CI-AKI, contrast-induced acute kidney injury; eGFR, estimated glomerular filtration rate; CHF, congestive heart failure; AMI, acute myocardial infarction; DM, diabetes mellitus; PCI, percutaneous coronary intervention.

In order to confirm the reliability of the study results, we performed several subgroup analyses that included patient age (whether over 75 years), gender (whether male), PCI status, and the existence of congestive heart failure, acute myocardial infarction, DM, hypertension, and other factors. Subgroup analysis is shown in Figure 6. Among the patients with eGFR ≥ 60 mL/min/1.73 m^2^, those with elevated SII level had a higher risk of CI-AKI. Both the SII-M and SII-H groups showed statistically significant differences compared to the SII-L group ([SII-M vs. SII-L]: OR = 1.19, 95% CI [1.05–1.36], *p* = 0.008; [SII-H vs. SII-L]: OR = 1.87, 95% CI [1.65–2.12], *p* < 0.001). By contrast, there was no statistically significant difference between the SII-M and SII-L groups in CAD patients with eGFR < 60 mL/min/1.73 m^2^ ([SII-M vs. SII-L]: OR = 1.22, 95% CI [0.98–1.51], *p* = 0.072), but a statistically significant difference was observed between the SII-H and SII-L groups ([SII-H vs. SII-L]: OR = 1.29, 95% CI [1.05–1.59], *p* = 0.018). Moreover, a significant interaction was found between eGFR and SII level in relation to the risk of CI-AKI (P for interaction = 0.005).

It is noteworthy that we found the phenomenon in subgroup analysis. When patients were at a better renal function level, there was a statistically significant difference in the risk of the SII-M group compared with the SII-L group. However, this difference disappeared when renal function was poor. In addition, a similar phenomenon was found in the subgroup analysis of age and congestive heart failure.

## Discussion

This multicenter, large-sample, retrospective cohort study indicated that elevated SII level was significantly correlated with an enhanced CI-AKI risk in CAD patients diagnosed with CAG. Our study further revealed a nonlinear relationship between SII and CI-AKI risk, with an inflection point at 19.12 × 10^11^/L. Below this inflection point, higher SII levels were associated with progressively increased risk of CI-AKI. However, SII did not significantly affect CI-AKI risk above the inflection point. Additionally, subgroup analysis revealed an interaction between eGFR and SII.

Mehran et al. [4] reported a CI-AKI prevalence ranging from 3.3% to 14.5%. Other previous studies have shown that the incidence of CI-AKI after coronary angiography and interventional therapy is higher (7.1–10.5% [21,22]), compared with computed tomography (2.4–6.4% [23,24]). Although intra-arterial administration is associated with a higher risk of CI-AKI compared with intravenous contrast, the exact underlying mechanism is unclear [25]. The incidence of CI-AKI in our study was 10.5%, similar to the prior research.

SII has been recognized as a sensitive inflammatory marker, and previous studies have shown its predictive capacity for CI-AKI [11,26,27]. Most previous studies investigating the relationship between SII and CI-AKI have focused on STEMI patients. For example, Öztürk et al. found that SII may be a promising inflammatory indicator for predicting contrast-induced nephropathy (CIN) after primary PCI in STEMI patients [12]. Karauzum et al. emphasized that, in contrast to NLR and PLR, SII may be a valuable and reliable marker for predicting CIN in STEMI patients [14]. Bağcı et al. proposed that the capacity of SII to predict CIN in STEMI patients after PCI is comparable to that of high-sensitivity CRP and exceeds that of NLR and PLR [13]. In contrast to previous studies focusing on STEMI patients, our research encompassed a broader population, thereby enhancing the clinical applicability of our findings.

Our team’s previous research by Lai et al. demonstrated that elevated SII before the CAG was associated with an increased risk of CI-AKI and pointed out that preoperative SII level may be an optimal monitoring indicator for preventing CA-AKI and prognosis [28]. Jiang et al. also investigated the predictive value of SII for CI-AKI in patients undergoing CAG, demonstrating that SII was most strongly associated with CI-AKI risk in comparison to NLR, PLT, and CRP, and it improved the predictive accuracy of CI-AKI [29]. They focused on postoperative populations following CAG. Notably, in addition to patients diagnosed with CAD, the studies also included a significant number of non-CAD patients. Similar to their findings, our research confirms that elevated preoperative SII level is associated with increased CI-AKI risk for CAD patients. Moreover, Our study distinguishes itself through several notable strengths. First, it focused on CAD patients, which strengthened the relevance of our findings to this particular population. Second, it featured a substantial sample size, providing robust statistical power for analysis. Third, the inclusion of multiple centers across China enhanced the diversity and representation of the study population. Fourth, we revealed a nonlinear relationship between SII and CI-AKI risk and analyzed it by using the threshold effect model. Fifth, we found the interaction between SII and eGFR level.

The pathogenesis of CI-AKI remains unclear, but inflammation and immune response play a critical role. Inflammation is the body’s defense mechanism against pathogens, injury, or other harmful stimuli, while excessive inflammation can lead to tissue damage and dysfunction [30]. During the occurrence of CI-AKI, the initiation and continuation of inflammatory response causes severe damage to renal tissue, and the possible mechanism between inflammation and CI-AKI can be speculated as follows.

First, Immune cell imbalance plays a crucial role in CI-AKI pathogenesis [31]. Iodinated contrast agents can induce tubular epithelial cell barrier disruption and endothelial cell injury and necrosis, strongly enhancing immune response and the release of inflammatory mediators [32]. This leads to neutrophil overactivation, further exacerbating tubular injury, increasing vascular permeability, and impairing endothelial function [26]. Additionally, neutrophil degranulation releases proteases and generates reactive oxygen species, worsening renal tissue damage [33]. Moreover, CD4^+^ T lymphocytes have been closely linked to CI-AKI, with their subgroups playing a key role in mediating immune responses [31]. Regulatory T cells (Tregs) provide renal protection with their anti-inflammatory properties [34]. Lymphocyte apoptosis weakens antioxidant defenses, aggravating injury [35–37]

Second, platelet activation and vascular thrombosis are critical mechanisms by which inflammation contributes to CI-AKI. The Inflammatory state can activate platelets through chemokines, secretory proteins, and microRNAs [38]. Inflammation is widely recognized as a prothrombotic state [39], with endothelial injury and inflammatory conditions creating a microvascular environment conducive to thrombosis, which leads to impaired renal blood perfusion, aggravates renal ischemia and hypoxia and further triggers local inflammatory response and kidney damage [40]. Furthermore, platelets can interact with neutrophils to form platelet-neutrophil aggregates (PNAs) [41], which induce neutrophil infiltration into tissues and release inflammatory mediators, exacerbating kidney damage through vascular injury and tissue destruction.

Third, NF-κB is a pivotal transcription factor involved in the inflammatory response, playing an important role in the regulation of immune and inflammatory pathways. In response to infection or other inflammatory stimuli, NF-κB becomes activated and enters the nucleus to regulate the transcription of target genes [42]. *In vitro* experiments have confirmed that contrast agents induce increased expression of high mobility protein B1 (HMGB1), activation of inflammatory receptors TLR2 and CXCR4, followed by a significant increase in NF-κB, and then the release of IL-6 and MCP-1, which leads to inflammatory factor expression and apoptosis [43]. Consistent with the above study, this was also confirmed in a rat model of another study. Inhibiting NF-κB phosphorylation prevented NF-κB from entering the nucleus and reduced renal IL-6 levels, which could reduce inflammatory response and oxidative damage and protect renal function in rats [44]. Therefore, the NF-κB pathway may be a potential target for the treatment of CI-AKI, and it is expected to attenuate the inflammatory response and renal injury by modulating its activity or inhibiting its function.

Strikingly, our study identified a nonlinear relationship between SII and the risk of CI-AKI in CAD patients undergoing CAG. Threshold effects analysis further quantified this nonlinear relationship. We speculate that below the inflection point (SII ≤ 19.12 × 10^11^/L), the inflammatory state reflected by SII may not yet reach saturation, and the increase in SII may reflect significant activation of inflammation and immune response. Therefore, within this range, the risk of CI-AKI continued to increase as the SII level rose, which suggested the effectiveness and reliability of SII as a CI-AKI risk management tool.

However, after the SII exceeded the inflection point (SII > 19.12 × 10^11^/L), there was no significant change in the risk of CI-AKI. We hypothesized the following causes of this phenomenon: (1) When SII exceeded the inflection point, the body was in a state of high inflammation, and the inflammatory state reflected by SII might tend to be saturated. Therefore, even if the SII level continued to rise, their exacerbating effect on the risk of CI-AKI may no longer be significant. In addition, compensatory protective mechanisms, like the release of anti-inflammatory factors, may be activated to counteract the excessive inflammatory response [34,45]. These anti-inflammatory mechanisms could partially neutralize the persistent inflammatory stimulus, reducing the impact of further increase in SII on CI-AKI risk. (2) Elevated SII level was frequently linked to chronic inflammation or related complications (e.g., diabetes or chronic kidney disease) [20,46,47]. This chronic inflammatory state may lead to a reduced sensitivity to acute inflammatory response; thus, although the SII level was elevated, it could reflect a chronic rather than acute inflammatory state, thereby weakening the ability to assess CI-AKI risk.

Furthermore, subgroup analysis revealed a significant interaction between SII and eGFR. We found that the early warning capability of SII is particularly relevant in patients with preoperative eGFR ≥ 60 mL/min/1.73 m^2^. Patients with eGFR ≥ 60 mL/min/1.73 m^2^ are generally considered to be likely to have a normal renal function or only mild decline in renal function. Interestingly, our study found that even among these patients with a moderate increase in SII (i.e., SII ≥ 3.74 × 10^11^/L), the risk of CI-AKI was significantly higher compared to those with lower SII levels. However, this phenomenon was not observed in patients with eGFR < 60 mL/min/1.73 m^2^. This difference may be partly due to higher underlying chronic inflammation levels, differences in metabolic status, and underlying medication use (e.g., SGLT2 inhibitors, statins, etc.) in patients with CKD, which may somewhat ‘mask’ the effects of moderate SII, with significant increases in risk only observed in the higher SII level. Based on this phenomenon, we speculated that severely impaired renal function may be a major determinant of CI-AKI risk in patients with eGFR < 60 mL/min/1.73 m^2^.

In comparison, SII may serve as a reliable biomarker for CI-AKI in patients with normal renal function or only mild decline in renal function. On the one hand, when considered alongside the eGFR level, SII could provide a more comprehensive and nuanced approach to the risk stratification for CI-AKI. On the other hand, the finding highlighted the importance and value of closely monitoring SII levels in patients with better renal function, as they are often overlooked in routine clinical assessments.

Analogously, we observed the similar phenomenon in the subgroups of age and CHF patients, revealing an interactive effect between SII level and different ages or cardiac function states in relation to the risk of CI-AKI, emphasizing the importance of considering these interdependent factors when assessing CI-AKI risk. Although our subgroup analysis uncovered these new findings, more studies are needed to elucidate the potential relationship.

In our study population, we unexpectedly found a relatively high prevalence of diabetes mellitus in the SII-M group, suggesting a possible association between diabetes mellitus and the immune-inflammatory state of the body. Previous studies have also shown that diabetes mellitus is often associated with metabolic disorders and chronic low-grade inflammatory processes [48], which may affect the number and distribution of peripheral blood leukocytes and platelets. The more pronounced chronic low-grade inflammatory response in the diabetic population may be a potential reason why it is more likely to be at moderate SII levels. In addition, this phenomenon does not exclude the possibility that there is an inherent difference in SII levels between diabetic and non-diabetic patients, leading to an increased prevalence of diabetes within the moderate SII group. On the other hand, the retrospective design of this study is not sufficient to infer a causal relationship between diabetes and SII. Although the increased prevalence of diabetes mellitus observed in the SII-M group provides an important clue to the association between the two, the impact of other potentially confounding factors (e.g., use of medications with potential effects on immune and inflammatory responses, basal metabolic status, severity of comorbidities, etc.) on the results still needs to be considered. These factors may go some way to explaining why diabetic patients are more inclined to have moderate levels rather than higher or lower SII levels. Therefore, we need to further explore the interaction between diabetes and immune-inflammatory markers (including SII) in a more systematic manner in prospective cohort studies or randomized controlled trials.

These findings constitute the unique strengths of our study and provide important guidance for future clinical practice. Based on our findings, we propose that SII has the potential to be a reliable risk management tool in future clinical practice for CAD patients undergoing CAG. Specifically, our study demonstrated that the risk of CI-AKI increased steadily with rising SII below the inflection point. This finding emphasized the substantial value of SII as an inflammation biomarker to identify high-risk individuals in the early clinical stage, which can assist in making proactive medical decisions and optimizing patient management. Future studies should aim to elucidate the underlying biological mechanisms and conduct cohort studies to evaluate the relationship between the dynamic changes of SII and CI-AKI. Furthermore, future studies should perform randomized controlled trials assessing the efficacy of anti-inflammatory therapies in the prevention of CI-AKI, including perioperative use of anti-inflammatory drugs such as sodium-glucose cotransporter-2 (SGLT-2) inhibitors [49] and colchicine. These pharmacologic interventions may offer an effective approach to modulate inflammatory responses and thereby reduce the incidence of CI-AKI.

Despite these worthwhile findings, our study has several limitations. Firstly, due to the nature of the retrospective cohort study, only associations could be drawn without the causal relationship being inferred. However, our study incorporated comprehensive data on potential major confounders, allowing for meticulous adjustment in the analysis. Secondly, our study did not account for comorbid conditions such as hematologic disorders or autoimmune diseases or the influence of treatments with corticosteroids or nonsteroidal anti-inflammatory drugs (NSAIDs). Additionally, we only examined preoperative SII levels and did not account for postoperative changes, which could present potential confounding effects. Further prospective cohort studies or randomized controlled trials are expected.

## Conclusion

This study demonstrates that preoperative SII may be an independent predictor of CI-AKI risk in CAD patients, and the eGFR level should also be considered. We identified a nonlinear relationship between SII and CI-AKI risk, with an inflection point at 19.12 × 10^11^/L. Threshold analysis revealed that, below this point, elevated SII significantly increased the CI-AKI risk; whereas, above the inflection point, the effect of SII on CI-AKI risk was no longer significant.
